# Oral administration of butylated hydroxytoluene induces neuroprotection in a streptozotocin-induced rat Alzheimer’s disease model *via* inhibition of neuronal ferroptosis

**DOI:** 10.1186/s10020-024-00980-y

**Published:** 2024-11-08

**Authors:** Parisa Faraji, Elham Parandavar, Hartmut Kuhn, Mehran Habibi-Rezaei, Astrid Borchert, Elham Zahedi, Shahin Ahmadian

**Affiliations:** 1 Institute of Biochemistry and Biophysics, University of Tehran, Tehran, Iran; 2 Department of Biochemistry, Charité - Universitätsmedizin Berlin, Corporate member of Freie Universität Berlin and Humboldt Universität zu Berlin, Charitéplatz 1, D-10117 Berlin, Germany; 3 School of Biology, College of Science, University of Tehran, Tehran, Iran; 4 Institute of Physiology, Tehran University of Medical Sciences, Tehran, Iran

**Keywords:** Neurodegeneration, Butylated hydroxytoluene, Oxidative stress, Cell death, Lipid peroxidation, Neuronal dysfunction, Lipoxygenase

## Abstract

**Background:**

Alzheimer’s disease (AD) is the most common human neurodegenerative disorder worldwide. Owing to its chronic nature, our limited understanding of its pathophysiological mechanisms, and because of the lack of effective anti-AD drugs, AD represents a significant socio-economic challenge for all industrialized countries. Neuronal cell death is a key factor in AD pathogenesis and recent studies have suggested that neuronal ferroptosis may play a major patho-physiological role. Since ferroptosis involves free radical-mediated lipid peroxidation, we hypothesized that enteral administration of the radical scavenger butylated hydroxytoluene (BHT) might slow down or even prevent the development of AD-related symptoms in an in vivo animal AD model.

**Material and methods:**

To test this hypothesis, we employed the rat model of streptozotocin-induced AD and administered butylated hydroxytoluene orally at a dose of 120 mg/kg body weight. Following BHT treatment, neuronal cell death was induced by bilateral stereotactic intraventricular injection of streptozotocin at a dose of 3.0 mg/kg body weight. Three weeks after surgery, we assessed the learning capabilities and the short-term memory of three experimental groups using the conventional y-maze test: (i) streptozotocin-treated rats (BHT pre-treatment), (ii) streptozotocin-treated rats (no BHT pre-treatment), (iii) sham-operated rats (BHT pre-treatment but no streptozotocin administration). After the y-maze test, the animals were sacrificed, hippocampal tissue was prepared and several biochemical (malonyl dialdehyde formation, glutathione homeostasis, gene expression patterns) and histochemical (Congo-red staining, Nissl staining, Perls staining) readout parameters were quantified.

**Results:**

Intraventricular streptozotocin injection induced the development of AD-related symptoms, elevated the degree of lipid peroxidation and upregulated the expression of ferroptosis-related genes. Histochemical analysis indicated neuronal cell death and neuroinflammation, which were paralleled by aberrant intraneuronal iron deposition. The streptozotocin-induced alterations were significantly reduced and sometimes even abolished by oral BHT treatment.

**Conclusion:**

Our data indicate that oral BHT treatment attenuated the development of AD-related symptoms in an in vivo rat model, most probably via inhibiting neuronal ferroptosis. These findings suggest that BHT might constitute a promising candidate as anti-AD drug. However, more work is needed to explore the potential applicability of BHT in other models of neurodegeneration and in additional ferroptosis-related disorders.

## Introduction

Alzheimer’s disease is the most frequent cause of adult dementia (Nichols et al. 2019) and two major histopathological hallmarks have previously been described for this disorder: (i) Extracellular aggregation of amyloid-beta (Aß) peptides derived from the amyloid precursor protein (APP), which leads to the formation of characteristic Aß plaques. (ii) Intra-neuronal aggregation of hyperphosphorylated tau proteins forming neurofibrillary tangles (NFT) (Lane et al. 2018). Although NFTs can also be found in other neurodegenerative diseases, they are particularly pronounced in AD. However, the pathogenesis of AD is much more complex and more recent investigations suggested that oxidative stress may play an important role (Sultana and Butterfield 2010). Oxidative stress induces neuronal dysfunction and initiates different types of cell death pathways, such as apoptosis (Ryter et al. 2007) and ferroptosis (Plascencia-Villa and Perry 2021).

Ferroptosis is an iron-dependent form of regulated cell death (Stockwell 2022; Chen et al. 2021). It involves iron-catalyzed peroxidation of membrane lipids that leads to defective membrane functions (Stockwell et al. 2017). Under normal conditions, reactive hydroperoxy lipids are rapidly reduced to the less reactive alcohols and different types of glutathione peroxidases (Seibt et al. 2019) catalyze this hydroperoxide reduction employing reduced glutathione as electron donor. However, when the reductive capacity of cells is compromised, hydroperoxy lipids accumulate intracellularly and undergo secondary decomposition reactions. These secondary reactions involve the intermediate formation of free radicals, which can induce oxidation of protein-bound amino acids, of DNA- or RNA-bound nucleotides and of polyenoic fatty acids bound in the membrane lipids (Lushchak 2014). These secondary oxidation reactions can be prevented or at least slowed down by radical scavengers (Haider et al. 2020).

**Fig. 1:**
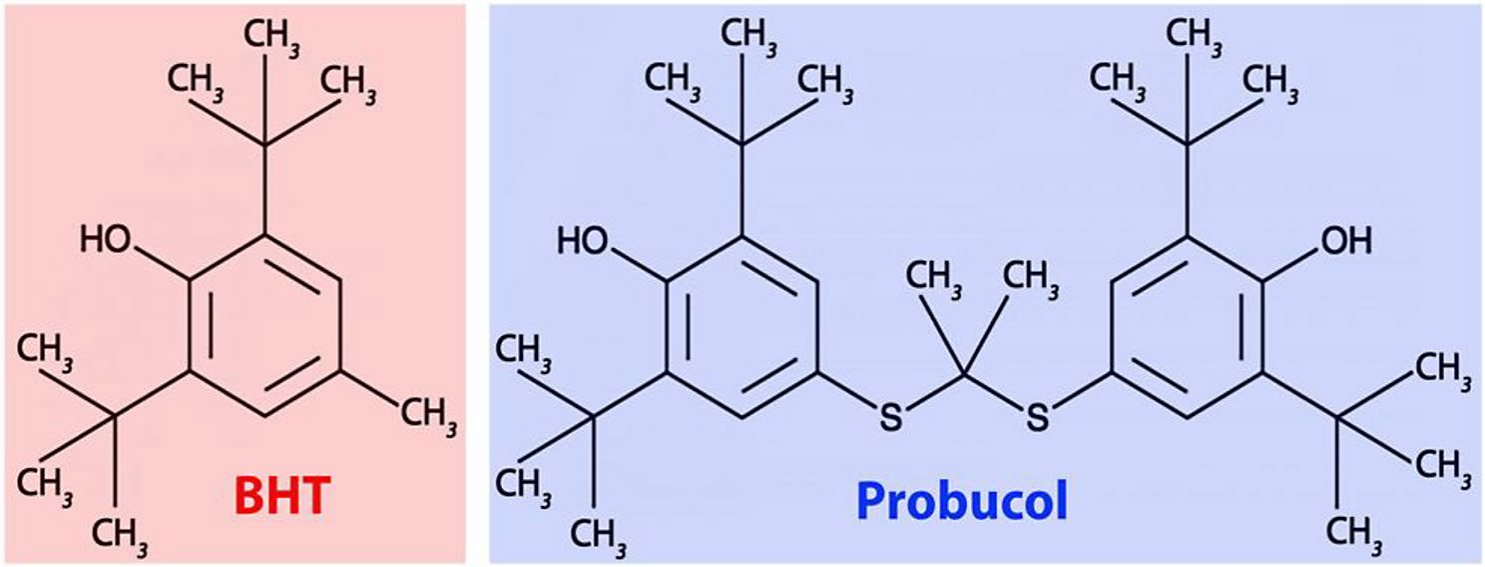
Structural comparison of BHT with the lipid-lowering drug probucol. Probucol basically represents a BHT dimer. The two monomers are covalently linked

Butylated hydroxytoluene (BHT, 2,6-di-*tert*-butyl-4-methylphenol) is such a lipophilic radical scavenger (Nieva-Echevarria et al. 2015; Williams et al. 1999). Its chemical structure resembles that of probucol (Fig. 1), a drug previously introduced for lipid-lowering therapy (Yamashita et al. 2021). The radical scavenging properties of the two compounds are related to their capability to delocalize the density of an additional electron within the aromatic system(s). BHT has previously been used as a food preservative since it prevents the peroxidation of food lipids (Nieva-Echevarria et al. 2015; Williams et al. 1999). It has a low acute toxicity (Yamamoto et al. 1980), but several studies suggested a possible link between BHT intake and cancer development (Thompson et al. 1991; Botterweck et al. 2000). Thus, despite its beneficial effects, the routine use of BHT in foods and pharmaceuticals is currently not encouraged. Similar dual activities have also been reported for BHT derivatives. For instance, sodium benzoate exhibits beneficial effects in the early-stages of AD (Piper and Piper 2017) but in mice it also impairs memory and induces oxidative stress (Khoshnoud et al. 2018).

We recently reported that BHT prevented RSL3- and ML162-induced ferroptosis in SH-SY5Y human neuroblastoma cells at concentrations as low as 30 nM (Faraji et al. 2024). Based on our observations we reasoned that RSL3 induces oxidation of cellular membrane lipids, which was prevented by pre-incubating the cells with BHT at sub-micromolar concentrations. Since neuronal ferroptosis has been implicated in the pathogenesis of AD (Wang et al. 2022; Zhang et al. 2022) we explored whether ferroptotic cell death might play a role in the streptozotocin-induced rat in vivo AD model and whether oral administration of BHT might slow down this process in vivo.

Neuronal ferroptosis has previously been implicated in the pathogenesis of AD (Wang et al. 2023; Huang 2023) but convincing experimental evidence for this hypothesis has neither been provided so far in animal in vivo AD models nor in human AD. In the present study, we employed the streptozotocin-induced rat AD model to explore whether oral administration of BHT might prevent the development of AD-related symptoms by inhibiting neuronal ferroptosis. Moreover, we show the principle in vivo activity of BHT to interfere with intracellular ferroptotic signaling and thus, this compound might also be tested in the future for its beneficial activities in other ferroptosis-related animal models of human diseases.

## Materials and methods

### Chemicals and devices

Reduced nicotinamide adenine dinucleotide phosphate (NADPH), reduced glutathione (GSH), oxidized glutathione (GSSG), glutathione reductase (GR), 5,5´-dithiobis-(2-nitrobenzoic acid) (DTNB), thiobarbituric acid (TBA), streptozotocin (STZ), butylated hydroxyl toluene (BHT), paraformaldehyde (PFA), hematoxylin and eosin (H&E) were purchased from Sigma–Aldrich (Darmstadt, Germany); ketamine, xylazine, phenylmethylsulfonylfluoride (PMSF), EDTA, EGTA, potassium phosphate and sodium azide were purchased from Chemicals Pvt. Ltd. (Tehran, Iran).

### Animals

For this study, we selected healthy male albino Wistar rats (8 weeks old), the body weight of which varied between 180 and 200 g. The rats were housed at the University of Tehran’s Central Animal House in the Department of Medical Physiology. Throughout the treatment and the surgical procedures, the rats were maintained under controlled environmental conditions including a room temperature of 25 ± 2 °C, a relative humidity varying between 45 and 55% and a 12-hour light/dark cycle. The animals had unrestricted access to standard rodent pellet diet and water. Before surgery, the rats underwent a fasting period of 12–18 h. All experimental procedures were conducted in strict compliance with the Animal Ethics guidelines of the University of Tehran, and the following certificate of allowance (IR.UT.SCIENCE.REC.1401.015, University of Tehran) was provided.

### Experimental design and research strategy

To explore the impact of oral BHT administration on the pathogenesis of AD, we employed the rat streptozotocin-induced in vivo AD model. This model has frequently been used in the literature (Akhtar et al. 2022; Silva et al. 2023; Duan et al. 2022) and involves stereotactic intraventricular application of streptozotocin. This treatment induces neuronal dysfunction, which is indicated by cognitive impairment and memory defects but also by oxidative stress, inflammation and neuronal cell death. Thus, the model mirrors important functional characteristics of human AD.

### Experimental groups

Three different groups of animals each involving 8 rats were set-up for the experiments. (i) Sham-group: These animals received an olive oil solution of BHT via tube feeding (120 mg/kg body weight) and underwent stereotactic surgery as explained below with 2.5 µL of PBS administered into each of the two lateral cerebral ventricles. (ii) AD-group: These animals received the same amount of olive oil (no BHT) as the sham group and they also underwent stereotactic surgery. As the BHT + AD group, the animals received 3 mg streptozotocin per kg body weight by stereotactic intraventricular injection. (iii) BHT + AD group: These animals received a BHT olive oil solution at a dose of 120 mg/kg body weight. They then underwent stereotactic surgery and received streptozotocin at a dose of 3 mg per kg of body weight.

### Oral administration of BHT

For exact dosing, an oily BHT solution (24 mg/mL in olive oil) was prepared and 0.5 ml of this solution was administered to each individual rat *via* a gastric tube. This procedure was repeated for four consecutive days. After 25 days, the animals underwent stereotactic surgery to administer intraventricularly streptozotocin as AD inducer.

### Intraventricular injection of Streptozotocin and post-operative care

Rats (eight weeks old, 180–200 g body weight) were anesthetized by an intraperitoneal injection of a mixture of ketamine (100 mg/kg body weight) and xylazine (10 mg/kg body weight). Then the animals were placed on a stereotaxic frame and the following coordinates (relative to bregma) were used for the intraventricular injections of the streptozotocin solution: anterior-posterior axis 0.9 mm, lateral axis 1.5 mm, dorso-ventral axis 4.0 mm. Intraventricular injections were carried out via a guide cannula with 27-gauge injection needles that were connected to a 5 µl Hamilton microsyringe. Animals received 3.0 mg streptozotocin per kg body weight (2.5 µL of a streptozotocin solution in PBS) as described previously (Chen and Zhang 2022). After surgery, the rats were kept in separate cages in a well-ventilated room at 25 °C. Food and water were placed inside the cage so that the animals could quickly recover from surgery.

### Y-maze test

Twenty days after stereotactic surgery, the Y-maze test was performed by an uninformed researcher (blinded research strategy). This test was developed to evaluate the spatial learning capabilities and the short-term memory functions of rodents (Kraeuter et al. 2019). For testing, a Y-shaped maze was used with three identical arms (60 cm of length) positioned at a 120° angle from each other. When the animal is placed in either of the three arms, it has free access to the two other arms. When an animal chooses a different arm than the one it came from this behavior is called an alternation. This choice is considered the correct response, whereas returning to the previous arm of the Y-maze is considered an error. The total number of arm entries and the sequence of entries were video recorded for calculation of the percentage of alternation. The movement of the animals was followed for 8 min and then the percentage of alternations was calculated as major readout parameter. After each test, the arms of the Y-maze were cleaned with 70% ethanol.

**Fig. 2:**
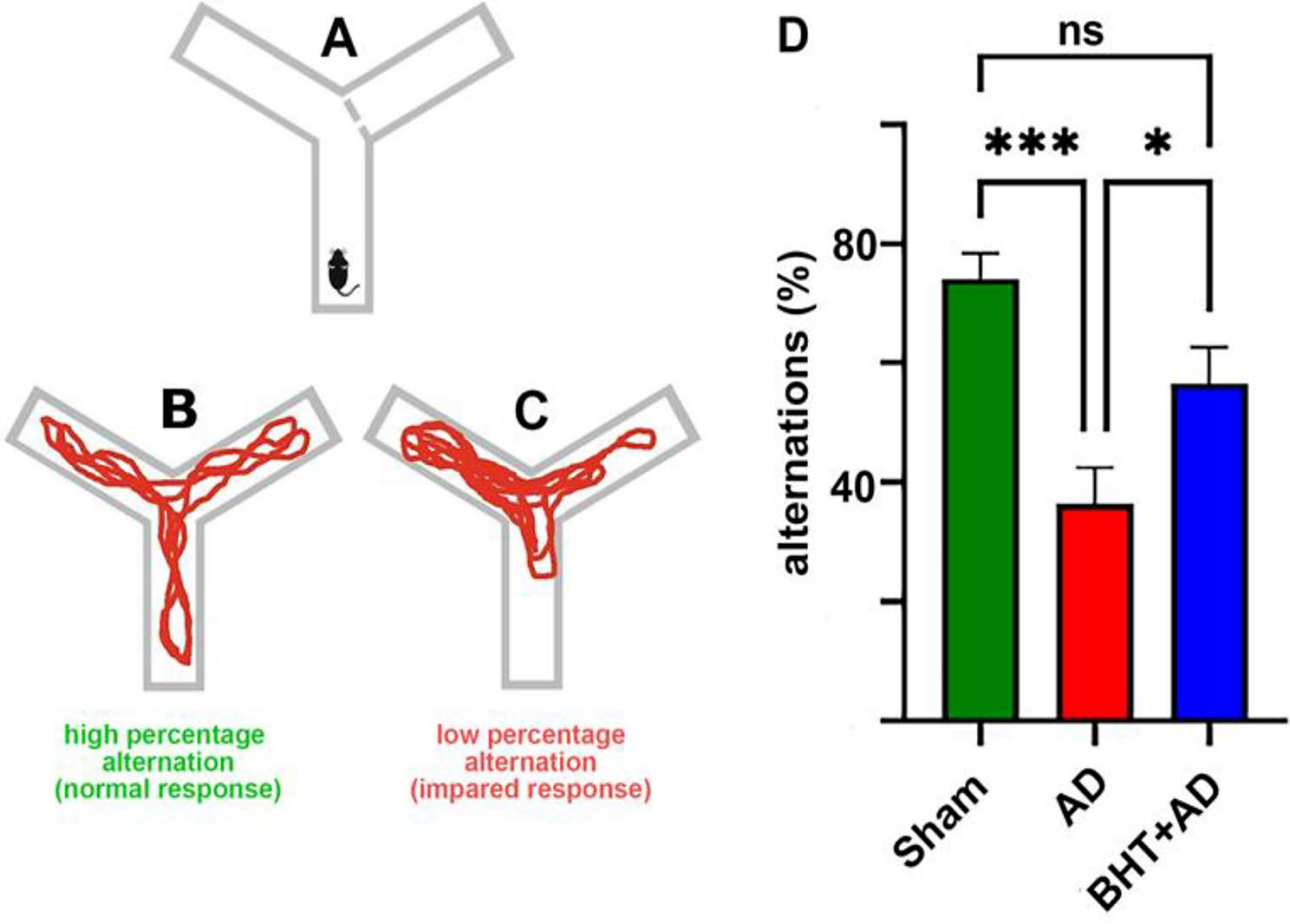
General principle of the Y-maze test and quantification of the percentage of alternations in rats of the three experimental groups. (**A**) The Y-maze test’s familiarization phase, with one arm closed. (**B**) Rats have normal orientation behavior. (**C**) Impaired orinetation behaviour of rats. (**D**) Quantification of individual alternations among the three experimental groups. Rats were pre-treated with BHT (BHT + AD group and Sham) or without BHT (AD group) and then received stereotactic intraventricular administration of streptozotocin (AD and BHT + AD groups) or of PBS (Sham group). After a recovery period of three weeks, all rats were tested once in the Y-maze, and the percentage of alternations was quantified for each rat. Eight individuals (*n* = 8) were used in each experimental group. ns, not significant; * *p* < 0.05; *** *p* < 0.001

For our experiments, a modified version of the Y-maze test was employed and our experimental protocol involved three consecutive steps: (i) Familiarization phase: During this phase, the animals were placed in one arm of the Y-maze and were allowed to freely explore two of the three arms of the maze. The entrance into the third arm was blocked (Fig. 2A). This phase serves to familiarize the animal with the device and the testing procedure. (ii) Retention phase: During this phase, the animals were returned to their home-cages for about 1 h. Here the rats did process the collected information and consolidated it. (iii) Test phase: Finally, the animals were reintroduced into the Y-maze, but this time all arms of the maze were freely accessible. Since rats have the natural tendency to explore novel environments, they preferentially enter the previously closed arm (novel arm) of the maze. In other words, entering the previously closed arm of the maze is the normal response (alternation). A high alternation frequency is normal (Fig. 2B) whereas reduced alternation frequencies suggest cognitive dysfunctions and/or memory defects (Fig. 2C).

### Preparation of brain tissue for biochemical analyses

Three weeks after stereotactic surgery, rats were sacrificed under anesthesia. The hippocampus was prepared, the tissue was snap-frozen in liquid nitrogen and stored at -80 °C. The tissue was homogenized at 4 °C in 10 mM Tris-HCl-buffer (pH 7.4) containing 2 mM phenylmethylsulfonylfluoride (PMSF) as a protease inhibitor as well as 10 mM EDTA and 0.1 mM EGTA as calcium-chelating compounds. The tissue homogenate was centrifuged at 1,000 g for 5 min at 4 °C and aliquots of the homogenate supernatants were used for MDA measurements, GPX activity assays and quantification of the GSH homeostasis.

### Measurement of malonyl dialdehyde

Malonyl dialdehyde (MDA) is a secondary product of lipid peroxidation and its tissue concentrations are considered a suitable measure to quantify the extent of oxidative challenge (Ayala et al. 2014). MDA can easily be quantified by its reactivity with thiobarbituric acid (TBA) and for our measurements we employed a TBAR detection kit (Kushanzist Azma, Tehran, Iran) that was used according to the instructions of the vendor.

### Activity assay of glutathione peroxidases

Glutathione peroxidases (Gpx) are anti-oxidative enzymes and the human genome involves nine different GPX genes (GPX1-9). In our studies, we quantified the total Gpx activity in the hippocampus homogenate supernatants. The reaction buffer was prepared as follows: To 100 µL potassium phosphate buffer (0.1 M, pH 7.0) we added 25 µL of an 0.1 M aqueous EDTA solution, 25 µL of aqueous sodium azide (10 mM), 25 µL of an aqueous glutathione reductase solution (2.4 IU/ml), 25 µL of an aqueous glutathione solution (0.01 M), 25 µL of an NADPH solution (1.5 mM dissolved in sodium bicarbonate). After mixing the ingrediants, 25 µL of the tissue homogenate supernatants were added, and the reaction mixture was incubated for 10 min at 37 ℃ in a quartz microtiter plate. Then the Gpx reaction was started by the addition of 25 µL of an aqueous H_2_O_2_ solution (1.5 mM) and the decrease in absorbance at 340 nm was recorded at room temperature. For quantification of the Gpx activity, a molar extinction coefficient for NADPH of 6.22 × 10^3^ M ^− 1^ cm ^− 1^ was used (Dixon et al. 2012).

### Determination of reduced glutathione (GSH) tissue concentrations

The tissue content of reduced glutathione (GSH) was quantified as described previously (Hirayama et al. 1983). In brief, 10 µL of tissue homogenate supernatants were mixed with 10 µL of 4% sulfosalicylic acid; the mixture was incubated at 4°C for one hour, and the protein precipitate was removed by centrifugation for 10 min at 4,000 g. 10 µL of the protein-free supernatant were added to 20 µL of a 5,5’-dithiobis-2-nitrobenzoic acid solution (4 mg/ml dissolved in 0.1 M Tris-HCl-buffer, pH 7). Then 270 µL of 0.1 M Tris-HCl buffer (pH 7) was added; the mixture was briefly vortexed and incubated at 25 °C for 15 min. The absorbance at 412 nm was assayed spectrophotometrically, and the tissue GSH content was quantified using a molar extinction coefficient of 13.6 × 10^3^ M^− 1^ x cm^− 1^ (Yang et al. 2016).

### Determination of oxidized glutathione (GSSG) tissue concentrations

The tissue content of oxidized glutathione (GSSG) was quantified using the Griffith’s method (Griffiths et al. 2014). For this purpose, 25 µL of tissue homogenate supernatant was transferred to the wells of a 96-well quartz microplate. Then 200 µL of reaction buffer was added, and the mixture was incubated for 10 min at 37 °C. The reaction buffer was previously prepared by mixing 100 µL 0.1 M Tris-HCl-buffer (pH 7.0) with 25 µL of an aqueous solution of glutathione reductase (2.4 IU/ml), 25 µl of an aqueous solution (10 mM) of NaN_3_), 25 µL of an aqueous solution of 0.01 M GSH, and 25 µL of an aqueous solution of NADPH (1.5 mM). In the final step, 25 µL of 1.5 mM H_2_O_2_ solution was added to the reaction mixture, and the sample was briefly shaken. The absorbance at 340 nm did immediately start to decrease, and the extent of this decrease was quantified by measuring the absorbance in 30-second intervals over a time period of 3 min using a microplate reader.

### Quantification of expression of AD-related genes using RT-PCR

To quantify the expression of AD-related genes by semi-quantitative RT-PCR, three animals from each experimental group were randomly selected. The brains were perfused with PBS, the hippocampus was isolated and total RNA was extracted using the RiboExTM RNA extraction kit (GeneAll Biotechnology, Seoul, Republic of Korea). The total RNA concentrations were quantified (A_260_/_280_ ratio) and reverse transcription was carried out using the reverse transcriptase kit of Parstus Biotechnolog (Tehran, Iran). Semi-quantitative RT-PCR was conducted for five AD-related gene products (*App*,* Gpx4*,* Fth1*,* Acsl4*,* Alox15*) and the internal reference gene Gapdh using the Power SYBRTM Green PCR Master Mix (Amplicon, Copenhagen, Denmark) and the Qiagen Rotor Gene device (Hilden, Germany). The amplification procedure involved an initial incubation of the amplification mixture at 50 °C for 2 min, followed by a denaturation step at 95 °C for 2 min. Next, 40 amplification cycles were carried out and each of these cycles consisted of a denaturation phase (15 s at 95 °C), an annealing phase (60 s at 60 °C) and an extension phase (60 s at 72 °C). The progress of gene amplification was monitored online and the amplification curves were evaluated using the ∆∆Ct method. The relative expression levels of the different target genes (*App*,* Gpx4*,* Fth1*,* Acsl4*,* Alox15*) were normalized for Gapdh expression. The primer sequences for cDNA amplification were as follows: *GPX4*: ATG TGT GCA TCC CGC GAT (forward), CAC GCA ACC CCT GTA CTT ATC C (reverse); *APP*: AAA ATG AAG TTG AGC CTG TCG (forward), GTT TGT CAA CCC AGA CCC TG (reverse); *Acsl4*: AAA ATG AAG TTG AGC CTG TCG (forward), GCC ACC GAT CAC AAT CTCA (reverse); *Alox15*: TGG CTG CAC CGT GGT TG (forward), CAG TTG CCC CAC CTG TAC AGA (reverse); *Fth1*: GCC AGA ACT ACC ACC AGG AC (forward), CGG TCA AAA TAA CAA GAC ATG G (reverse); and *Gapdh*: GGT GAA GGT CGG TGT GAA C (forward), TTG TCA CAA GAG AAG GCA GC (reverse). The PCR primers were synthesized by SinaClon (Teheran, Iran).

### Histopathological examinations

#### Preparation of microscopic sections

For histological analysis hippocampal tissue was prepared and small pieces were fixed in 4% paraformaldehyde (PFA) for 72 h. Then the tissue samples were transferred to a 30% sucrose solution for cryoprotection. Finally, serial cryosections with a thickness of 10 μm were cut and mounted on cryoslides (Suchorukova et al. 2010). For paraffin embedding the tissue was first immersed in a 4% PFA solution to preserve the cellular structure. After fixation, the tissue was dehydrated in graded ethanol solutions followed by xylene treatment. Then the tissue was infiltrated with liquid paraffin wax and embedded in a specific orientation in a paraffin block. Thin sections, typically 5 to 10 μm thick, were cut using a microtome and mounted on glass slides. Later on, the cross-sections were stained in different ways and were finally inspected under the microscope (Zeiss, Dublin, CA, USA).

#### H&E staining

Initially the tissue sections were stained using the standard hematoxylin and eosin (H&E) protocol. For this purpose, the paraffin-embedded sections were incubated at 60 °C to extract the paraffin. Following deparaffinization, the sections were immersed in xylol (xylene) to ensure complete removal of the paraffin. After xylene treatment the sections were gradually rehydrated by incubating them in solutions with decreasing ethanol concentrations (100%, 95%, 70%) and then they were finally rinsed with distilled water. The sections were subsequently stained with hematoxylin solution for 14 min followed by thorough washing with water for 3 min. Then the sections were dipped in 10% acetic acid three times and rinsed again with water. Next, the sections were stained with eosin for 5 min. After eosin staining the sections were again dehydrated and finally treated with xylene. The stained sections were then prepared for microscopic analysis (Feldman and Wolfe 2014; Fischer et al. 2008).

#### Congo-red staining

Congo-red staining is commonly used to detect Aß peptides in formalin-fixed and paraffin-embedded tissue sections. In Congo-red stained sections Aß deposits appear red, while nuclei are counterstained in blue. For this type of staining paraffin-embedded sections were first de-parafinized at 60 °C and then the sections were rehydrated by soaking them in solutions with decreasing concentrations of ethanol in water (100%, 95%, 70% etc.). Finally, the sections were washed with distilled water. Then the sections were stained with a 1% Congo-red solution for 60 min and were washed with distilled water for 5 min. After rinsing the slides were quickly dipped (5–10 times) in an alkaline ethanol solution (1% sodium hydroxide in 50% ethanol) and washed again in water. Subsequently, the slides were counterstained with hematoxylin for 30 s and immersed in ammonia containing water for 30 s until the slices turned blue. After washing with water the slides were dehydrated again by incubating them in aqueous solutions with increasing ethanol concentrations (Mariam 2020; Bennhold 1922).

#### Nissl staining

Nissl staining is frequently used to selectively stain neuronal cell bodies. Following de-paraffinization and rehydration the slides were stained using 1% cresyl violet solution and sections were covered with Antlan glue. Then, the sections were washed with tap water, dehydrated with alcohol and treated with xylene. Finally, the sections were inspected under a microscope (Zeiss, Dublin, CA, USA) at 10-fold magnification and morphologically intact neurons were counted. For cell counting the CA1 region of the hippocampus was selected and the number of morphologically intact-looking neurons in this region was quantified (Azad et al. 2011).

#### Perls staining

3,3’-diaminobenzidine (DAB) enhanced Perls’ staining was used to quantify the intracellular iron levels (Serrano-Pozo et al. 2011). For this purpose, paraffin-embedded brain sections were de-paraffinized at 60 °C and rehydrated as described above. The sections were then soaked in distilled water for 5 min and subsequently incubated in freshly prepared Perls’ solution (5% potassium ferrocyanide / 5% hydrochloric acid) for 30 min. After intensive washing with PBS, endogenous peroxidase activity was blocked by incubation of the slides in a 3% hydrogen peroxide solution. Following this, the slides were immersed for 3 min in a 0.05% DAB solution (Sigma-Aldrich, Darmstadt, Germany) for counterstaining (Falangola et al. 2005).

### Data presentation and statistical analysis

Data are presented as means ± SD (*n* = 3–8). The number of biological and technical replicates for each experiment is specified in the legends of the figures and the tables. Statistical analysis of the raw data was carried out with a one-way ANOVA test using the Prism software (version 8.3.1.). This software package was also used to structure the bar diagrams. A p-value < 0.05 was considered statistically significant for all test systems. For preparation of the images the Adobe Photoshop program (version 21.1.1.) was used.

## Results

### Intraventricular application of streptozotocin induced learning and memory defects but BHT prevented these functional in vivo alterations

The Y-maze spontaneous alternation test quantifies the willingness of rodents to explore new environments. Typically, animals prefer to investigate a new arm of the maze (Fig. 2A-C) rather than returning to the one they have previously visited (alternations). Functional changes in hippocampal neurons affect the number of alternations and thus, a decrease in the alternation percentage indicates a loss of neuronal function in the hyppocampus. In our study, we used the Y-maze test to quantify the degree of hippocampal malfunction induced by intraventricular injection of streptozotocin.

As indicated in Fig. 2D, sham-operated rats (negative controls) exhibited an alternation percentage of about 75%, which is normal for rats of this age. However, after intraventricular administration of streptozotocin (AD group), the percentage of alternations decreased to about 35%, with a statistically significant difference between the Sham and the AD groups (*p* < 0.001). When rats received BHT (BHT + AD group) before streptozotocin treatment the alternation percentage increased to about 50% showing a significant difference to the AD group (*p* < 0.05). In contrast, the alternation percentage between the Sham-operated and BHT + AD groups was not significantly different. These data suggest that the animals of the BHT + AD group develop less severe streptozotocin-induced hippocampal neuronal malfunctions when compared with the AD group.

### Intraventricular streptozotocin administration induces oxidative stress in the hippocampus that is prevented by BHT

Lipid peroxidation plays an important role in ferroptosis (Dodson et al. 2019; Tang et al. 2021) and polyunsaturated fatty acids (PUFAs) are suitable substrates for both enzymatic and non-enzymatic lipid peroxidation. There are several ways to determine the degree of lipid peroxidation, but in this study we quantified the formation of thiobarbituric-reactive substances (TBARS) as primary readout parameter.

**Fig. 3:**
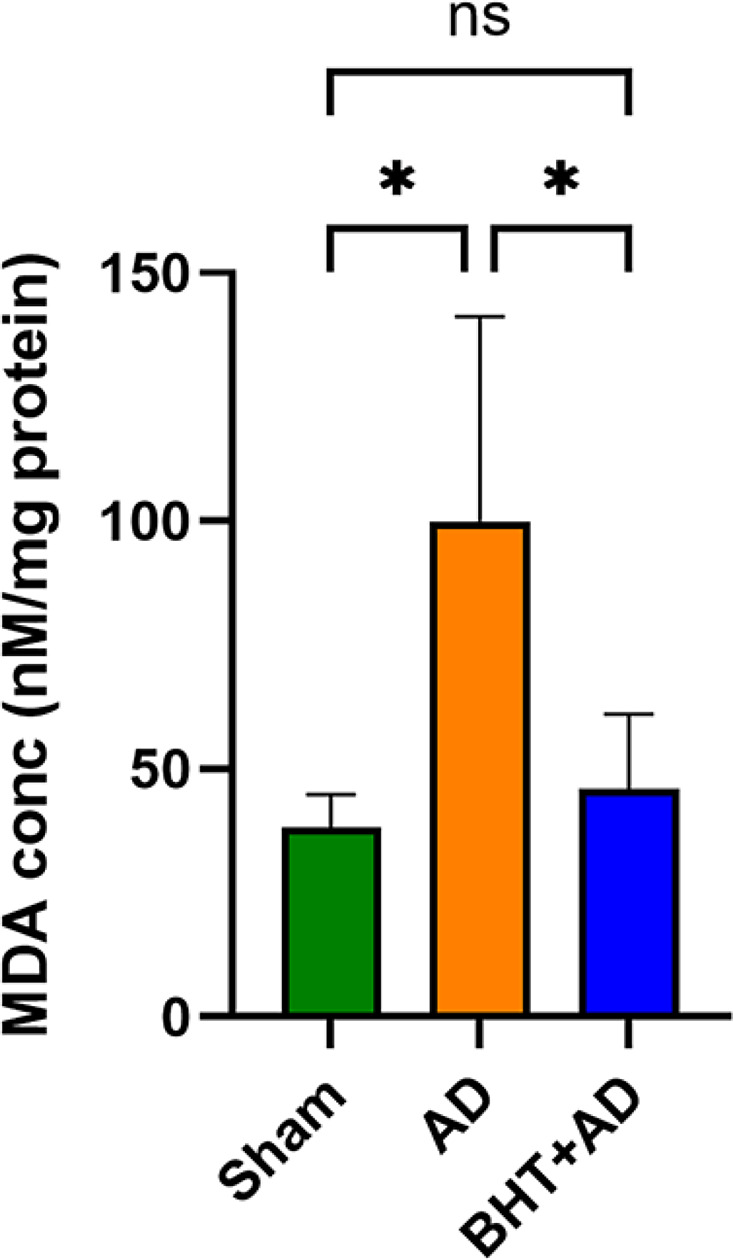
Qunatitication of TBARS as readout parameter for the degree of lipid peroxidation in the hippocompus of streptozotocin-treated rats. Intraventricular streptozotocin injection induced the formation of AD-related symptoms. After the recovery period, rats were sacrificed, the hippocompus was preared, the tissue was homogenized, and aliquots of the homogenate supernatants were tested for TBARS. The amounts of TBARS are given in nM malonyl diadehyde equivalents per mg homogenate supernatant protein. Each experimental group (column) is represented by three different rats (three biological replicates), and each homogenate supernatant was measured in dublicate (two technical replicates). * *p* < 0.05; ns, not significant

Figure 3 shows that streptozotocin administration (AD group) significantly elevated the levels of TBARS in the hippocampus compared to Sham-operated animals (no streptozotocin injection). Interestingly, oral administration of BHT almost completely prevented this increase (BHT + AD) and reduced the MDA concentrations to control (Sham) levels. It should be stressed at this point that TBARS represent an array of different secondary lipid peroxidation products and that the amounts of TBARS might not adequately mirror the degree of primary lipid peroxidation.

### Determination of glutathione peroxidase (GPX) catalytic activity

Ferroptotic signaling involves inhibition of the Xc-system, which functions as an amino acid antiporter within the plasma membrane of mammalian cells. It exchanges intracellular glutamate for extracellular cystine at a 1:1 molar ratio (Yang and Stockwell 2016). The imported cystine is subsequently reduced to cysteine, a critical substrate for the biosynthesis of glutathione (GSH). Consequently, the Xc-system is of major importance for anti-oxidative defence.

**Fig. 4:**
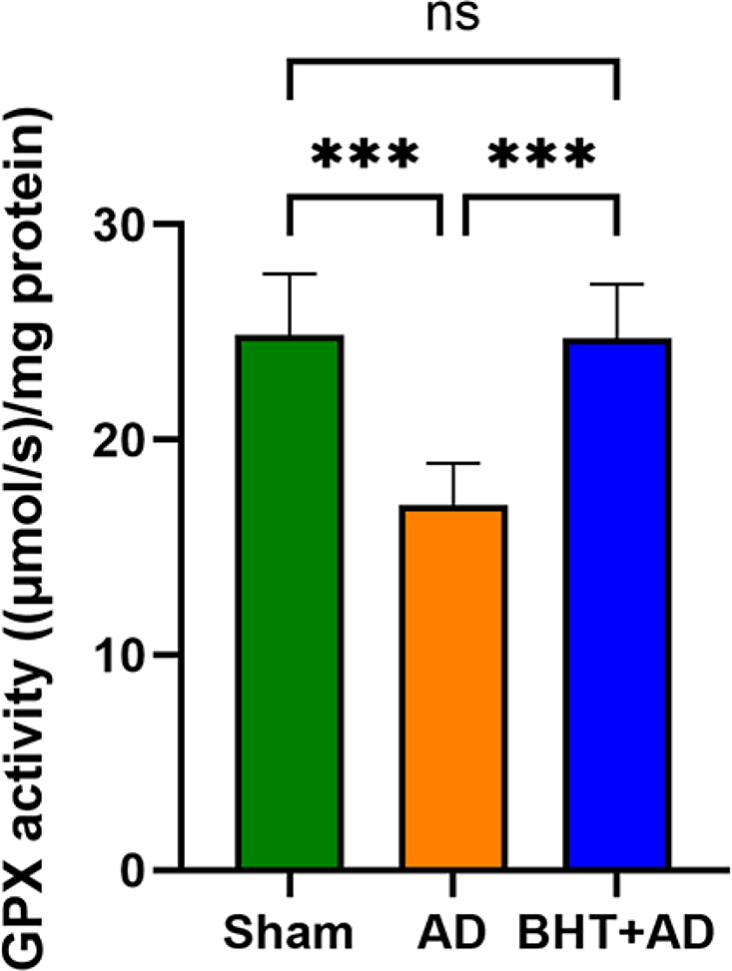
Quantitation of the catalytic activity of Gpx isoforms in tissue homogenate supernatants of the hippocampus of streptozotocin-treated and control rats. Intraventricular streptozotocin was used to induce AD-related symptoms. After the recovery period, rats were sacrificed, the hippocampus was preared, the tissue was homogenized, and aliquots of the homogenate supernatants were tested for Gpx activity using H_2_O_2_ as substrate. Each experimental group (column) is represented by three different rats (three biological replicates), and each homogenate supernatant was measured in dublicate (three technical replicates). *** *p* < 0.001; ns, not significant

Since the catalytic activity of glutathione peroxidases (Gpx) depends on the availability of reduced glutathione (GSH), inhibition of the Xc-system downregulates the cellular anti-oxidative capacity, which might lead to an accumulation of intracellular peroxides and thus, to ferroptosis (Stockwell 2017, 2022; Dixon et al. 2012). When we quantified the GPX activity in the homogenate supernatants of the hippocampus (Fig. 4), we found a significantly (*p* < 0.001) reduced catalytic activity of the Gpx isoforms after intraventricular administration of streptozotocin (AD group) compared with Sham-operated animals. This reduction was not observed when the rats were orally pre-treated with BHT (BHT + AD group). These data suggested that BHT protected rats from the streptozotocin-induced loss of the catalytic activity of Gpx-isoforms. The human genome involves nine different Gpx genes and each of them encodes for a special Gpx-isoform. Some of these isoforms, in particular Gpx4, are more important for ferroptotic signaling than others. In this study we did not selectively quantify the activity of Gpx4 but the total Gpx activity in hippocampal tissue.

### Quantification of the glutathione homeostasis

The glutathione / glutathione disulfide ratio (GSH/GSSG ratio) is a suitable readout parameter to characterize the cellular redox state (Wu and Batist 2013; Rae and Williams 2017). In intact cells, oxidized GSSG is continuously reduced back to GSH by glutathione reductase (Couto et al. 2016), which utilizes NADPH as an electron donor. NADPH mainly originates from the oxidative pentose phosphate pathway (Stincone et al. 2015) but can also be produced by the malic enzyme or other transhydrogenases (Liu et al. 2017).

**Fig. 5:**
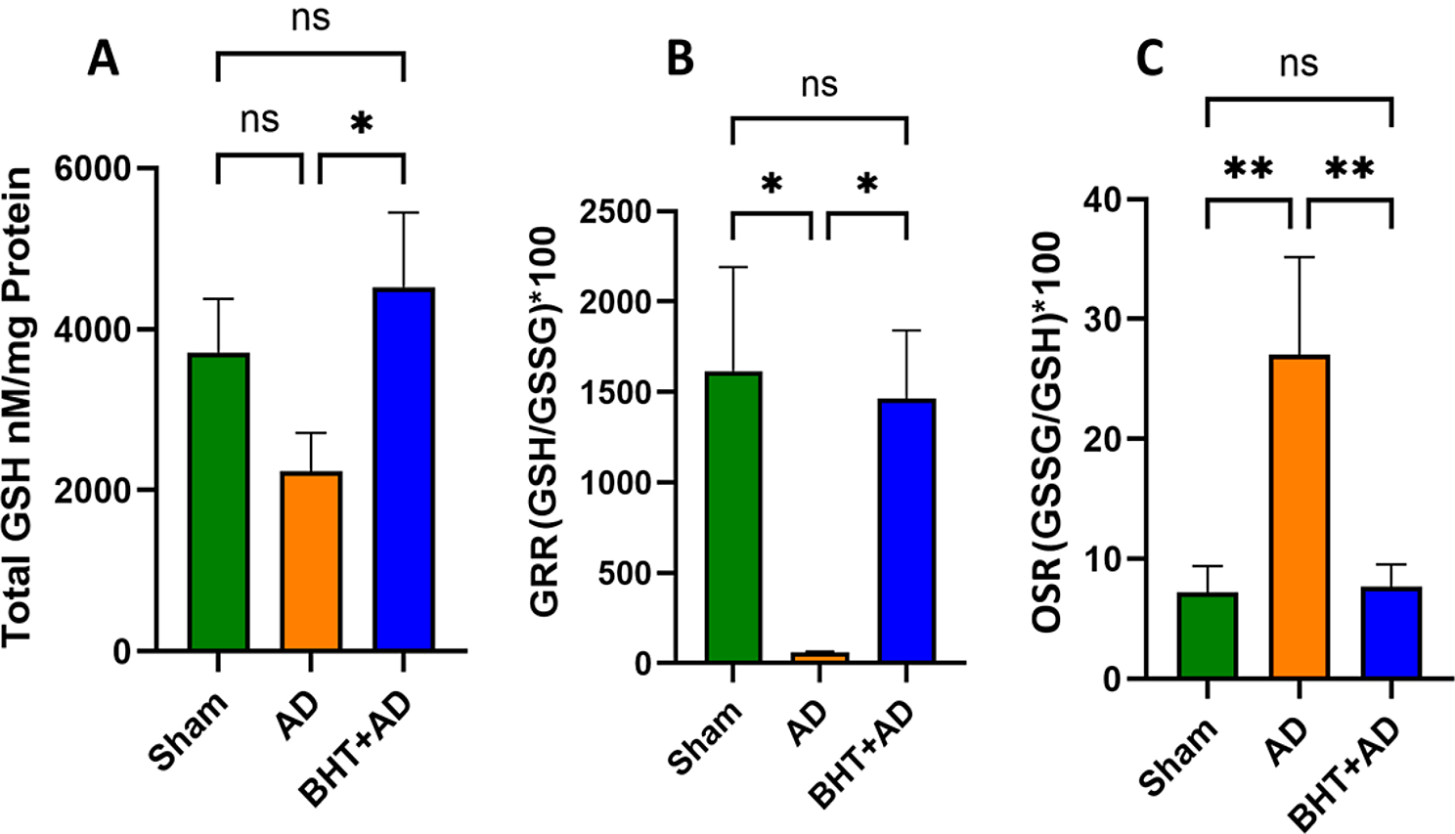
Glutathione homeostasis changes in the hippocampus of streptozotocin-treated rats and corresponding control animals. Intraventricular streptozotocin was used to induce AD-related symptoms. After the recovery period, rats were sacrificed, the hippocompus was prepared, the tissue was homogenized, and aliquots of the homogenate supernatants were tested for total GPX activity using H_2_O_2_ as substrate. Each experimental group (column) is represented by three different rats (three biological replicates) and each homogenate supernatant was measured in duplicate (three technical replicates). **A**) Absolute GSH tissue concentrations. Panel **B**: Glutathione reduction rate (GRR, GSH / GSSG ratios). **C**) Oxidative stress ratio (OSR, GSSG, / GSH ratios). * *p* < 0.05; ** *p* < 0.01; ns, not significant

Although alternative electron donors are available for various Gpx isoforms, reduced GSH is the most commonly used reductant for this class of anti-oxidative enzymes (Brigelius-Flohe and Maiorino 2013). When intracellular GSH levels drop the catalytic activity of Gpx isoforms, which controls the cellular peroxide level, is likely to decline. To explore whether intraventricular administration of streptozotocin modifies the GSH-GSSG equilibrium, we first quantified the GSH and GSSG levels in the homogenate supernatants of the hippocampus and then calculated the glutathione redox ratio (GRR, GSH/GSSG) and the oxidative stress ratio (OSR, GSSG/GSH) as suitable readout parameters for the oxidative challenge of the effected brain region. As shown in Fig. 5A, the hippocampal GSH levels in the AD group were lower than those of Sham-operated animals, and the difference was statistically significant (*p* = 0.05). However, when streptozotocin-treated rats were orally pre-treated with BHT, the drop in GSH levels was prevented, and we observed significantly higher GSH levels in the BHT + AD rats than in the AD animals. On the other hand, no difference was detected when the GSH levels of the Sham group were compared with those of the BHT + AD rats.

Although the absolute cellular GSH levels are a suitable parameter for quantifying the degree of oxidative stress, the glutathione redox ratio (GRR) and the oxidative stress ratio (OSR) are more informative. Figure 5B shows that streptozotocin-treated rats (AD group) exhibited significantly reduced GRRs when compared with Sham-operated animals (Sham group). Oral pre-treatment with BHT prevented the GRR reduction, and the GRR values of the BHT + AD group were similar to those detected in Sham-operated animals. In other words, oral BHT administration prevented the streptozotocin-induced drop in the cellular GSH/GSSG ratios.

Finally, we also quantified the OSR (GSSG/GSH quotient) in the tissue homogenate supernatants, and the data shown in Fig. 5C confirm the results of GRR quantification. Here we found that streptozotocin treatment (AD group) induced a strong increase in OSR when compared with Sham-operated animals (Sham group). However, oral BHT pre-treatment prevented the increase in OSR, and there was no statistical difference between Sham-operated animals and BHT + AD rats.

Taken together, our data quantifying the glutathione homeostasis confirmed the previous conclusion that intraventricular stretozotocin administration induced oxidative stress in the hippocampus of rats and that oral pre-treatment with BHT almost completely prevented these alterations.

### Histopathological examinations

Extracellular deposition of Aß proteolysis products (Aß plaques formation) is a histopathological hallmark of AD (Lane et al. 2018; Serrano-Pozo et al. 2011; Khan et al. 2020) and Congo red staining can visualize Aß plaques in microscopic brain sections  (Wisniewski et al. 1989).

**Fig. 6:**
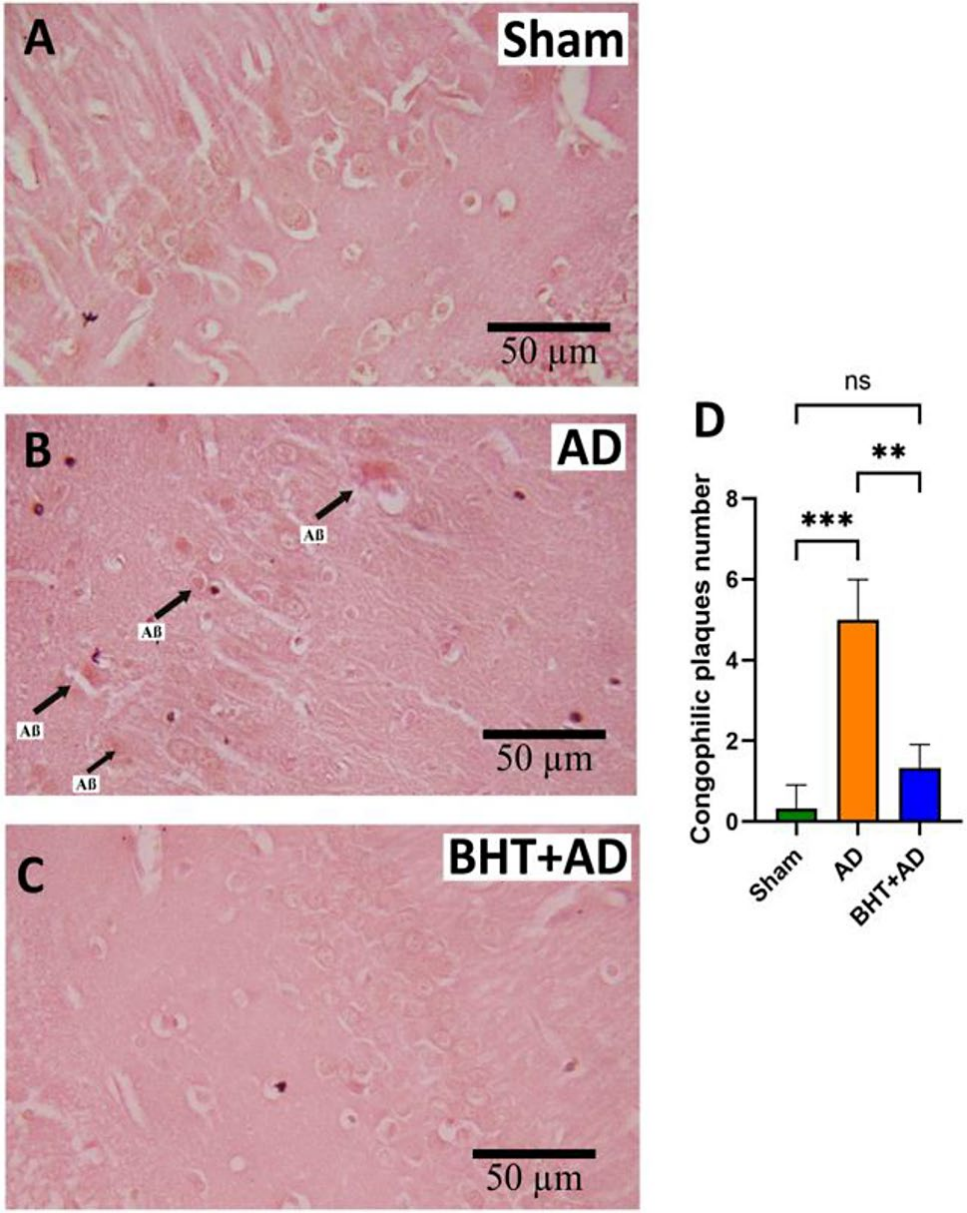
Congo-red staining of micropscopic cross-sections of the hippocampus. Intraventricular streptozotocin was used to induce AD-related symptoms. After the recovery period, rats were sacrificed, the hippocompus was isolated, and microscopic cross-sections were prepared and stained using the Congo red method (see Materials and methods). Staining of Aß plaques is indicated by the black arrows. Each experimental group (column) is represented by three different rats (three biological replicates) and from each individual, three different slides were randomly selected from each individual for quantification (panel D). Representative images for the three different experimental groups are shown. **A**) Sham-operated rats (no streptozotocin treatment, **B**) Sterptocotocin-treated rats (no BHT tretment), **C**) Sterptocotocin-treated rats that were orally pre-treated with BHT. **D**) Quantification of Congo red staining. Errors indicate morphological alterations

When we stained serial sections of the hippocampus of Sham-operated animals using the Congo red method (see Materials and methods) we did not observe significant staining of Aß plaques (Fig. 6A). In contrast, a specific staining pattern was observed when sections were prepared from the hippocampus of streptozotocin-treated rats (Fig. 6B). Oral BHT pre-treatment largely prevented Aß plaque formation (Fig. 6C) since we detected minimal Congo red-positive staining in corresponding sections from BHT + AD rats. In Fig. 6D, Congo red staining was quantified, and these data suggest that streptozotocin administration induced Aß plaque formation, which was largely prevented by BHT pre-treatment of the animals.

**Fig. 7:**
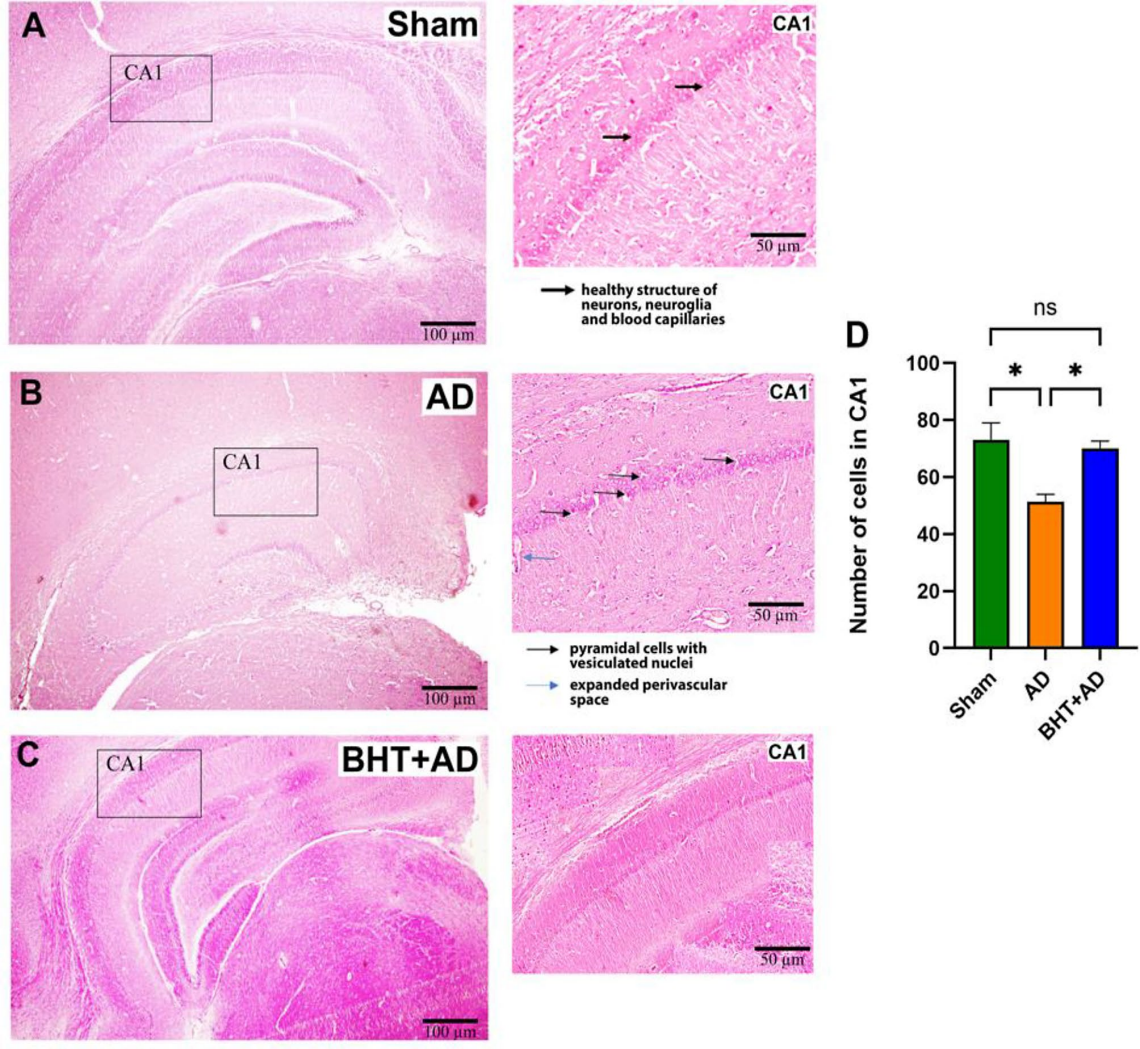
Standard H&E staining of representative micropscopic sections of the CA1 area of the hippocampus. Intraventricular streptozotocin was used to induce AD-related symptoms. After the recovery period, rats were sacrificed, the hippocompus was isolated, and microscopic sections were prepared and stained using the conventional H&E method (see Materials and methods). Panel A: **A** representative image of a sham-operated rat. The thick black arrows identify normal-looking pyramidal cells. Panel **B**: Representative image of a streptozotocin-treated rat. Pyramidal cells with vesiculated nuclei. **C**) Representative image of a streptozotocin-treated rat that was previously pre-treated with BHT. No major structural defects were observed. **D**) Quantification of intact-looking neurons per 100 µm^2^. Each experimental group (column) is represented by three different rats (three biological replicates) and from each individual, three different slides were randomly selected for quantification. Data represent means ± SD. *p < 0.05; ns, not significant. Errors indicate morphological alterations

AD is a neurodegenerative disease that induces structural alterations in neurons and glial cells (McGeer et al. 2006). Moreover, the inflammatory reaction induces neuronal cell death and standard H&E staining can be used to quantify cell numbers (neurons and glial cells). On the other hand, this technique does also allow quantification of morphologically aberant cells. When we inspected H&E-stained sections of the hippocampus from the three different experimental groups, we predominantly observed intact-looking neurons and glial cells in Sham-operated animals (Fig. 7A). Moreover, the blood capillaries appeared regularly structured, with no signs of inflammatory vasodilatation (Fig. 7A). In contrast, in streptozotocin-treated rats (AD group) a large number of pyramidal cells were visible, which involve vesiculated nuclei as signs of neurodegeneration (Fig. 7B). Moreover, the perivascular space was conspicuously widened, and these changes suggest severe neuroinflammation. In BHT-treated animals (Fig. 7C), these alterations were not detected, suggesting that oral pre-treatment of rats with BHT largely prevented neurodegeneration and neuroinflammation. Since neurodegeneration reduces the number of normal-looking neurons, we finally counted the numbers of normal-looking neurons in the microscopic sections and found a significant reduction in the AD animals (Fig. 7D). This reduction was completely prevented when the animals were pre-treated with BHT. Taken together, these results emphasize that BHT has a promising potential for mitigating the structural abnormalities associated with streptozotocin-induced neurodegeneration.

**Fig. 8:**
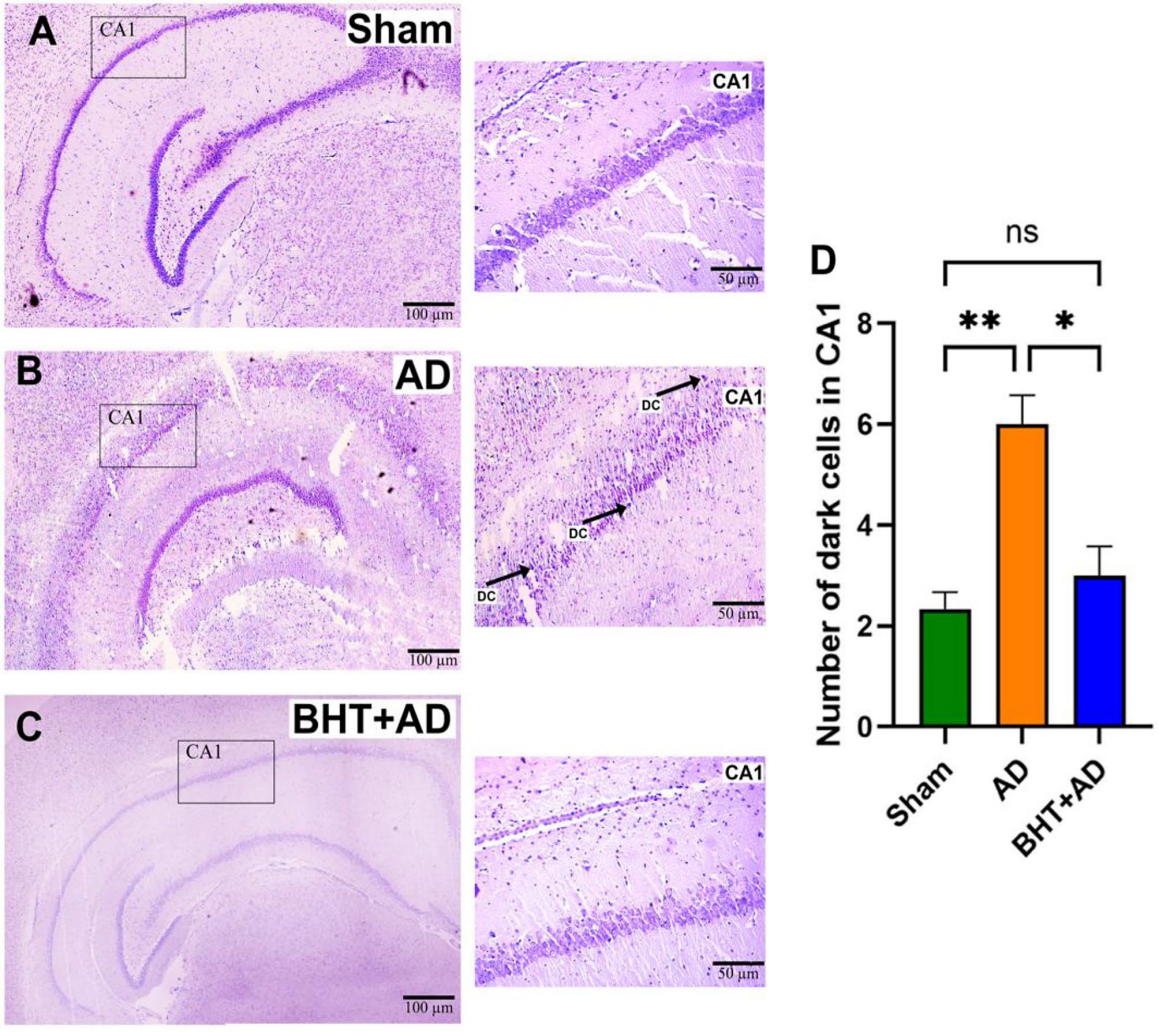
Nissl-staining of micropscopic sections of the hippocampus. Intraventricular streptozotocin was used to induce AD-related symptoms. After the recovery period, rats were sacrificed, the hippocompus was isolated, and microscopic cross-sections were prepared and stained using the Nissl-method (see Materials and methods). Dysfunctional neurons (darkly stained) are indicated by arrows. Panel **A**: Representative image of a Sham-operated rat (Sham group). Panel **B**): Representative image of a streptozotocin-treated rat (AD group). DC – dark cells. The dark color of these cells is caused by iron overload. **C**) Representative image of a streptozotocin-treated rat that was pre-treated with BHT (BHT + AD group). **D**) Quantification of dysfunctional (darkly stained) neurons per 100 µm^2^. Each experimental group (column) is represented by three different rats (three biological replicates) and from each individual, three different slides were randomly selected for quantification. Data represent means ± SD. *p < 0.05; ** *p* < 0.01; ns, not significant; DC = dark cell

Since H&E staining is relatively unspecific, we employed a more neuron-specific staining method (Nissl staining) to confirm the basic conclusions drawn from standard H&E staining. As shown in Fig. 8A + D, the CA1 region of the hippocampus of Sham-operated rats exhibited a regular structure with a relatively low number of darkly stained dysfunctional neurons (no arrows). After streptozotocin administration (Fig. 8B + D) the regular structure was disturbed, and more dysfunctional cells (darkly stained cells, DC) were observed (arrows). Most interestingly, these morphological alterations were prevented when the rats were pre-treated with BHT (Fig. 8C + D).

From Fig. 7 + 8 it was concluded the intraventricular streptozotocin administration reduced the number of functional neurons in the CA1 area of the hippocampus. The most plausible explanation for this observation is that streptozotocin administration induced premature cell death, but it has not been studied in detail, which kind of cell death (necrosis, apoptosis, ferroptosis) may prevail. Since neuronal ferroptosis has been implicated in the pathogenesis of AD (Bao et al. 2021) and since excessive intracellular iron accumulation is a key feature of ferroptosis (Li et al. 2021), we carried out Perls staining to quantify cells with iron overload.

**Fig. 9:**
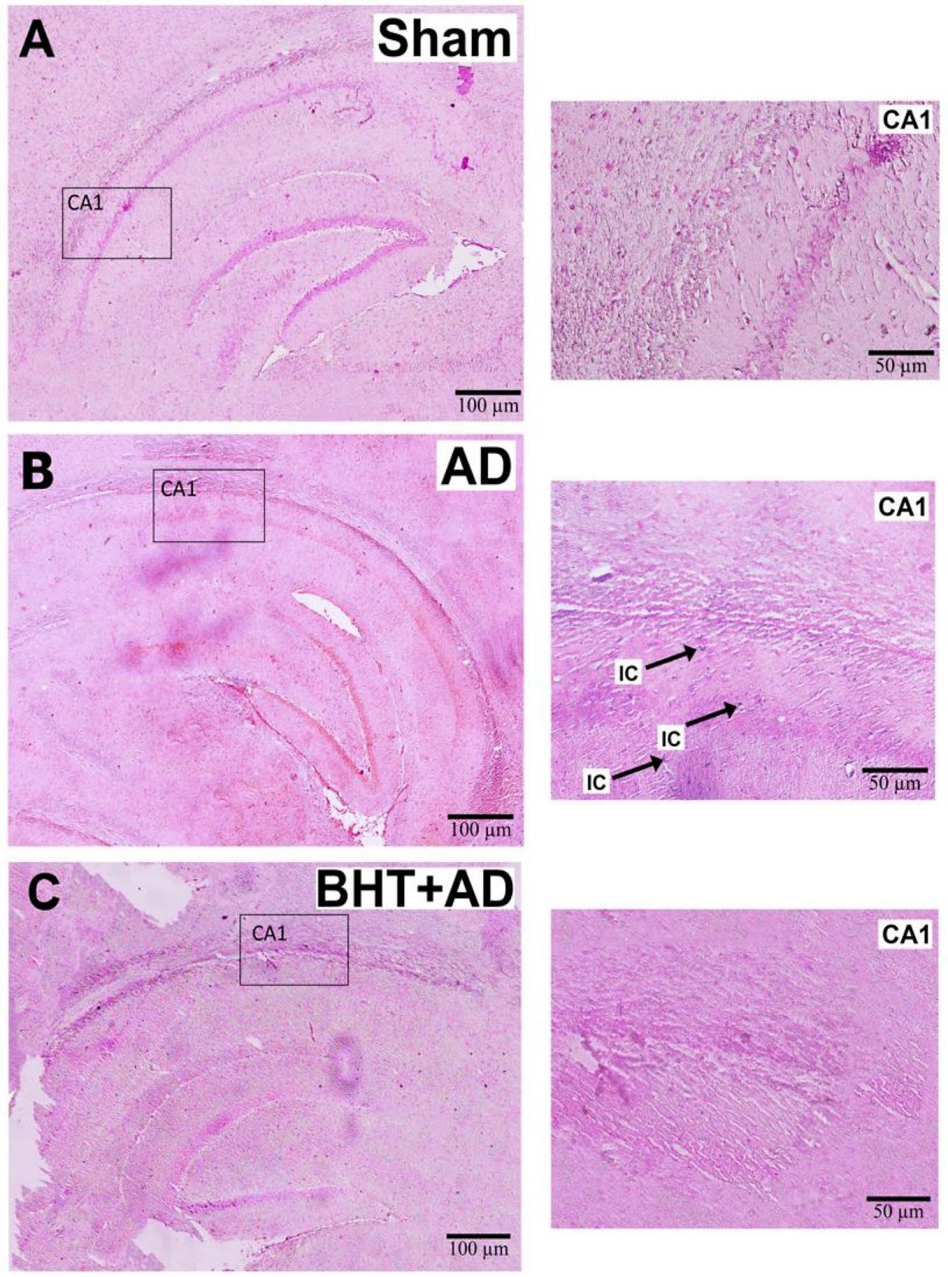
DAB-enhanced Perls’ staining of micropscopic sections of the CA1 area of the hippocampus. Intraventricular streptozotocin was used to induce AD-related symptoms. After the recovery period, rats were sacrificed, the hippocompus was isolated, and microscopic cross-sections were prepared and stained using the DAB-enhanced Perls-method (see Materials and methods). Cells with iron overload are indicated by arrows. Panel A: A representative image of a sham-operated rat (Sham group). Panel B): A representative image of a streptozotocin-treated rat (AD group). IC indicates cells with iron overload. Cells with a high iron content have an ICated with BHT (BHT + AD group)

In Sham-operated animals (Fig. 9A) we did not detect Perls-positive areas, and this data suggests that under these experimental conditions, cells were not overloaded with iron. In contrast, when cross-sections of streptozotocin-treated animals were stained (AD group) Perls-positive foci (arrows) were detected (Fig. 9B). When the animals were pretreated with BHT, a lower percentage of iron-overloaded cells were observed (Fig. 9C). These data suggest that BHT counteracts streptozotocin-induced intracellular iron accumulation under our experimental conditions. It should be stressed at this point that Perls’ staining identifies cells with iron overload but the staining procedure is not really quantitative. In other words, negative Perls’staining does not automatically mean that the intracellular iron concentrations are normal and thus, the relative share of cells with dysfunctional iron metabolism might be underestimated.

### Expression of AD- and ferroptosis-related genes is increased after streptozoticin administration and these changes are prevented by systemic BHT pre-treatment of the animals

In our experiments, we found that intraventricular streptozotocin administration induced memory and learning defects (Fig. 2), oxidative stress (Figs. 3–5), extracellular deposition of Aß cleavage peptides (Fig. 6), neuronal cell death (Fig. 7 + 8) and excessive intracellular iron deposition (Fig. 9). These findings indicate that this rat model adequately mirrors important pathophysiological processes of human AD. Since human AD also involves pronounced alterations in cerebral gene expression patterns (Chowdhury and Rajkumar 2020; Gatt et al. 2019), we next quantified the expression of AD-related and ferroptosis-related genes in the three different experimental groups.

For this purpose, total mRNA was extracted from the hippocampus of Sham-operated rats (Sham group), from streptozotocin-treated rats (AD group) and from streptozotocin-treated rats that had been orally pre-treated with BHT (BHT + AD group). After reverse transcription, the expression of AD- and ferroptosis-related genes [amyloid precursor protein (App), ferritin (Fth1), fatty acid CoA ligase 4 (Acsl4), glutathione peroxidase 4 (Gpx4), arachidonate 15-lipoxygenase (Alox15)] was quantified by RT-PCR. The results of these experiments, which are summarized in Table 1, indicate that all genes follow a similar expression pattern. Gene expression was low in Sham-operated animals (Sham group), but was significantly increased following streptozotocin administration (AD group) and the differences reached the level of statistical significance (*p* > 0.05). When the rats were pre-treated with BHT (BHT + AD group), expression levels were significantly reduced, reaching the expression levels of the Sham-operated animals. Taken together, our expression data suggest that the expression of AD- and ferroptosis-related genes is upregulated in this in vivo Alzheimer’s disease model and that this upregulation is prevented by oral administration of BHT.

**Table 1:** Cerebral expression of AD- and ferroptosis-related gene products

Group	Relative gene expression levels (%) and statistic significances									
	App gene		Gpx4 gene		Fth1 gene		Acsl4 gene		Alox15 gene	
	Expres- sion (%)	*p*- value	Expres- sion (%)	*p*- value	Expres- sion (%)	*p*- value	Expres- sion (%)	*p*- value	Expres- sion (%)	*p*- value
SHAM	100 ± 62.1	-	100 ± 27.6	-	100 ± 54.9	-	100 ± 7	-	100 ± 20.4	-
AD	173 ± 23.2	< 0.05^a)^	16 ± 7.1	< 0.05^a)^	647.5 ± 74.1	< 0.01^c)^	1230 ± 27.3	< 0.001^e)^	384 ± 13.7	< 0.05^g)^
BHT + AD	30 ± 6.0	> 0.05^b)^	54 ± 29.8	> 0.05^b)^	72.5 ± 18.7	< 0.01^d)^	20 ± 3.4	< 0.001^f)^	110± 11.1	> 0.05^h)^

AD-related symptoms were induced by intraventricular administration of Streptozotocin. After the recovery period rats were sacrificed, the hippocampi were prepared and total RNA was extracted. Following reverse transcription, the steady state concentrations of the selected mRNA species were quantified by RT-PCR using gene specific primer combinations. Expression of the target genes was normalized to *gapdh* expression and the mean expression level of the sham-operated group was set to 100%. For each experimental group (sham, AD, BHT + AD), three different rats (three biological replicates) were used and each RNA extract was measured in duplicate (two technical replicates). ^(a)^ SHAM vs. AD; ^(b)^ AD vs. BHT + AD; ^(c)^ SHAM vs. AD; ^(d)^ AD vs. BHT + AD; ^(e)^ SHAM vs. AD; ^(f)^ AD vs. BHT + AD; ^(g)^ SHAM vs. AD; ^(h)^ AD vs. BHT + AD

## Discussion

### Degree of novelty and advancement of science

**Fig. 10:**
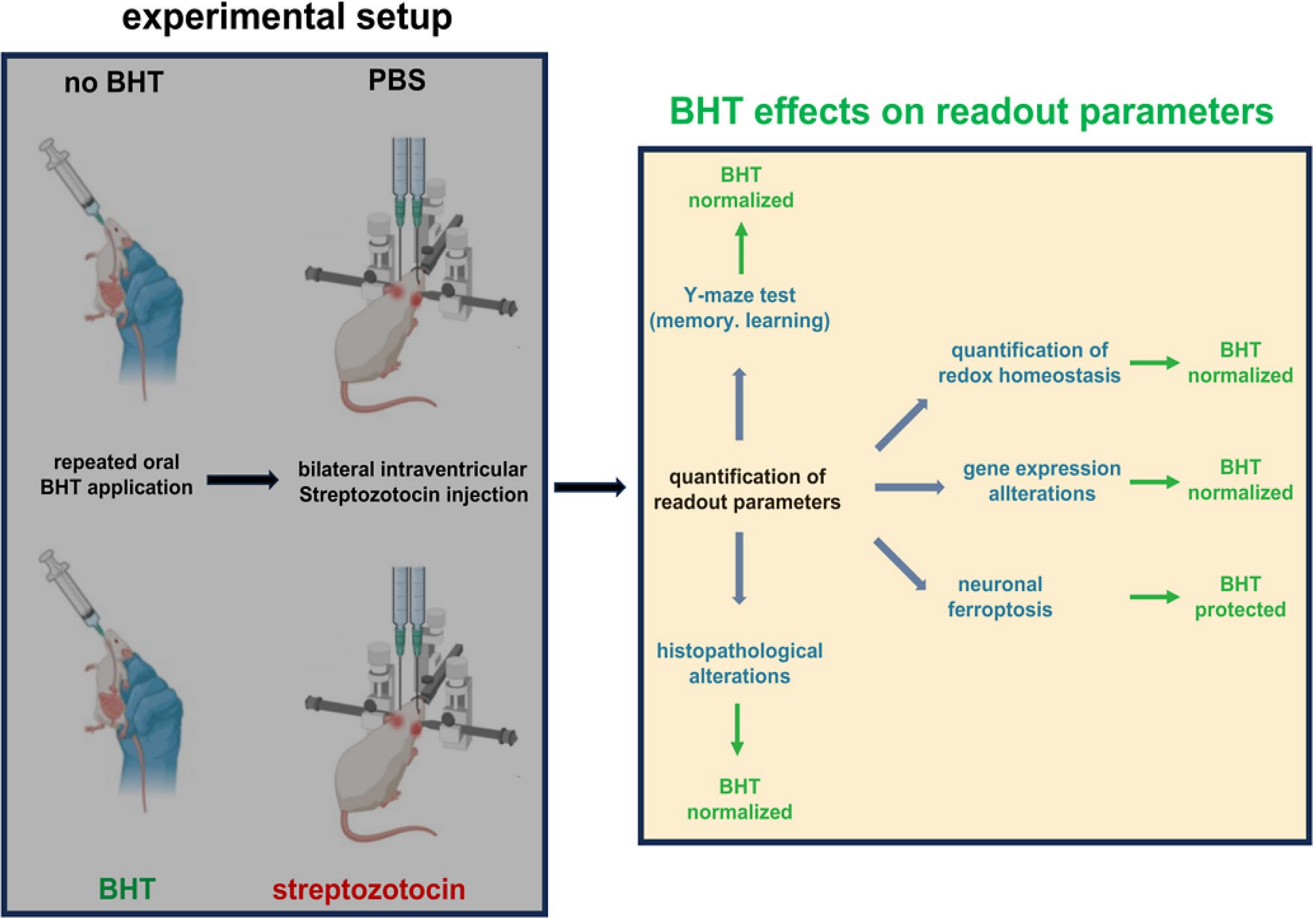
Experimental setup and beneficial effects of oral BHT administration in the streptozotocin-induced rat in vivo AD model. Left site: Experimental setup of the streptozotocin-induced rat AD model. More detailed explanations are given in the Materails and Methods section. Right site: The beneficial effects of BHT administration on different structural and functional readout parameters are shown in green

AD is the most prevalent neurodegenerative disease in all industrialized countries (Lane et al. 2018) and the formation of extracellular amyloid plaques and intraneuronal aggregation of hyperphosphorylated tau proteins are histological hallmarks of this disease (Serrano-Pozo et al. 2011). However, the pathogenesis of AD is much more complex and oxidative stress may play a patho-physiological role (Sultana and Butterfield 2010). In this study, we explored the impact of oral administration of the strong antioxidant BHT on different structural and functional readout parameters in an in vivo rat AD model (Fig. 10) and found that BHT normalized the deleterious consequences of intraventricular streptozotocin administration. Thus, in our animal model BHT exhibited neuroprotective effects.

Anti-oxidative therapy using naturally occurring and/or synthetic antioxidants might exhibit protective effects. For instance, in a cellular AD model, various oxazole-4-carboxamide/butylated hydroxytoluene hybrids inhibited glycogen-synthase-kinase 3β and reduced Tau-protein phosphorylation in HEK293 cells at sub-micromolar concentrations (Luo et al. 2023). These compounds alleviated H_2_O_2_-induced oxidative stress, modified the mitochondrial membrane potential, reduced Ca^2+^ influx and prevented neuronal cell death. Moreover, the most optimized oxazole-4-carboxamide/butylated hydroxytoluene hybrid (KWLZ-9e) prevented learning and memory impairment in an in vivo AD model (Luo et al. 2023). Although these compounds exhibit anti-oxidative properties it remains unclear of whether or not this property is responsible for the observed beneficial anti-AD effects.

Extracts of different parts of the monkey biscuit tree (*Piliostigma thonningii*) also exhibit anti-oxidative properties and such extracts have frequently been used in traditional medicine to manage cognitive impairment. Although the mechanistic details of these effects have not been studied in detail aqueous extracts of different parts of *P. thonningii* exhibited beneficial activities and reduced the formation of cerebral TBARS (Moriasi et al. 2020). Unfortunately, so far no detailed toxicity studies have been carried out and thus, at the moment the clinical use of such extracts cannot be recommended (Moriasi et al. 2020).

Probucol, which structurally resembles BHT (Scheme 1), was originally developed as antioxidative compound to prevent the degradation of tire rubber. However, later on it was applied as lipid-lowering drug in hypercholesterolemic patients (Yamashita et al. 2021). In Japan it has been used for a long time in lipid-lowering therapy, but in Western countries, its application was abandoned because it significantly reduces serum HDL-cholesterol (Yamashita et al. 2015). More recently, it has been shown that probucol might offer substantial potential for prevention and treatment of neurodegenerative diseases. A large number of preclinical studies have indicated that probucol exhibits anti-oxidative and anti-inflammatory activities (Sharif et al. 2024) but it still has not been proven that these beneficial effects are a functional consequence of its anti-oxidative properties.

### Effectiveness of anti-oxidative treatment in other AD models and in ferroptosis

In the present study we reported that anti-oxidative therapy might induce beneficial effects in a single in vivo animal AD model but it remains to be shown whether BHT might exhibit similar protective effects in other animal AD-models such as transgenic animals (Kang et al. 2021). Interestingly, anti-oxidative therapy with crocin prevented the formation of AD related symptoms in a cellular AD model (HT22 cells) but also under in vivo conditions. Here BHT treatment reduced the expression of the App gene (Wang et al. 2019).

Neuronal ferroptosis has previously been implicated in the pathogenesis of AD (Zhang et al. 2021; Jakaria et al. 2021) and oxidative stress constitutes a major hallmark of ferroptosis (Dixon and Stockwell 2019). Antioxidants reduce the degree of oxidative stress and thus, may interfere with ferroptotic signaling (Kuang et al. 2020). For instance, the natural product artemisinin, which can be extracted from the plant *Artemisia annua*, exhibits anti-oxidative properties and ameliorates the cognitive decline by inhibiting hippocampal neuronal ferroptosis and oxidative stress in mice (Wang et al. 2024). Similarly, various diphenylamine derivatives reduce oxidative damage and prevent neuronal ferroptosis (Hinder et al. 2021). Here again, the corresponding compounds clearly exhibit anti-oxidative properties but their mechanism of action has not been explored in detail. It may still be possible that the anti-oxidative properties of these compounds may not be the major reasons for the observed beneficial effects.

Glutathione peroxidases (Gpx), in particular Gpx4, are key elements in ferroptotic signaling since these enzymes control the cellular hydroperoxide levels. We found that intraventricular administration of streptozotocin elevated the degree of lipid peroxidation (Fig. 3) and reduced the hippocampal Gpx activity (Fig. 4). Since oral administration of BHT prevented these effects our data are consistent with the working hypothesis that oxidative stress may play a major role in both AD-related neuronal ferroptosis (Jankauskas et al. 2023) and AD-related memory defects (Li et al. 2022).

Iron is an essential trace element for all mammalian cells and the intracellular iron concentrations are tightly regulated. Cellular iron overload can trigger ferroptosis (Yan and Zhang 2019) and iron overloaded neurons have been detected in the brains of transgenic AD mice (Becerril-Ortega et al. 2014). In our study we quantified the abundance of iron overloaded neurons in the hippocampus of normal rats but did not find significant amounts of such cells. However, following streptozotocin-treatment Perls-positive neurons were detected in the hippocampus and BHT pre-treatment prevented this effect (Fig. 9). Ferritin is an important protein of the neuronal iron homeostasis and our data (Table 1) indicate that streptozotocin treatment strongly augmented the expression of the ferritin heavy chain (*Fth1*). In a transgenic mouse model of human AD ferritin accumulated in the surrounding Aβ plaques (Svobodová et al. 2019) and membrane lipid peroxidation can be attenuated by blocking Fth1 expression (Gong et al. 2022).

ACSL4 is a critical protein in intracellular ferroptotic signaling and its overexpression induces ferroptosis I (Yuan et al. 2016). In a mouse model of human AD suppression of Ascl4 expression mitigated the development of AD symptoms (Zhu et al. 2022). Consistent with this finding we observed that intraventricular streptozotocin administration elevated Ascl4 expression but oral BHT administration abolished this effect (Table 1).

### BHT is not an effective ALOX15 inhibitor but may prevent peroxide decomposition

ALOX15 is a lipid peroxidizing enzyme (Ivanov et al. 2015; Schewe et al. 1981), which has been implicated in ferroptotic cell death (Wenzel et al. 2017; Kagan et al. 2017). Since ferroptosis is involved in the pathogenesis of AD, ALOX15 might also be of pathophysiological relevance. In the brain ALOX15 is not well expressed in neurons. However, it was identified in infiltrating inflammatory cells but also in oligodendrocytes and in the microglia of ischemic brain regions (Haynes and Leyen 2013). Lipoxygenase isoforms have also been implicated in the pathogenesis of AD (Joshi et al. 2015; Chen et al. 2020), but the detailed pathophysiological functions of these enzymes in neurodegeneration remain unclear. Here we found that intraventricular streptozotocin application upregulated the expression of Alox15 by a factor of 4 and this regulatory response was partly prevented when rats were pre-treated with BHT (Table 1). However, our expression kinetics do not really prove a patho-physiological function of Alox15 in the pathogenesis of AD. To explore this topic more detailed studies employing Alox15^−/−^ mice should be performed.

Despite its antioxidant properties BHT is not a direct ALOX15 inhibitor. The IC_50_ for linoleic acid oxygenation by recombinant human ALOX15 is > 100 µM and thus, it is 2–3 orders of magnitude higher than those for other ALOX15 inhibitors (Kakularam et al. 2022). When different types of biomembranes (mitochondrial membranes, erythrocyte ghosts) were used as substrates for pure native rabbit ALOX15 the oxygenation of the membrane lipids was even augmented by BHT as indicated by an increased oxygen consumption and by a strongly elevated formation of specific oxygenation products (Schnurr et al. 1995). Although the molecular basis for this activating effect is still a matter of discussion it has been suggested that membrane binding of BHT might induce structural alterations in the membrane architecture, which may lead to a better susceptibility of the polyenoic fatty acid for lipoxygenase attack. In other words, BHT improves the substrate behavior of membrane lipids for ALOX15 attack. On the other hand, as antioxidant BHT might prevent secondary decomposition of ALOX15 derived hydroperoxy lipids. These secondary reactions involve the formation of free radicals that can be trapped by BHT. Thus, radical induced secondary oxygenation reactions are prevented by BHT (Schnurr et al. 1995).

The human genome involves six functional ALOX genes, which encode for six different ALOX isoforms. Unfortunately, BHT is not an effective inhibitor for any of them. On the other hand, specific inhibition of the pro-inflammatory ALOX5 slowed down the rate of neurodegeneration in a Parkinson disease model (Li et al. 2023) suggesting a patho-physiological role for this enzyme. However, a potential role of other ALOX isoforms (ALOX15B, ALOX12, ALOX12B, ALOXE3) in AD has not been explored.

### Limitations of the study

Although our experimental data suggest that oral treatment of rats prevents the development of AD-related symptoms, it should be stressed that the study has several limitations. The protective effect of BHT was only tested in a single rat AD model (intraventricular streptozotocin injection) and it remains unclear whether the observed protective effect can be confirmed in other animal AD models, particularly in genetically modified mouse AD models.

Human AD is an age-related disease and its pathogenesis is rather complex (Lane et al. 2018; Zhang et al. 2022). In general, animal models usually mirror certain aspects of a given human disease. Unfortunately, in most cases such models do not adequately describe the complexity of human diseases. In other words, it must be studied in the future whether the protective effect of BHT can be confirmed in other in vivo AD models. We have recently reported (Faraji et al. 2024) that BHT protects SH-SY5Y human neuroblastoma cells from RSL3- and ML162-induced ferroptotic cell death. Unfortunately, although SH-SY5Y cells are of neuronal origin they do not mirror important functional characteristics of differentiated human neurons. Thus, it remains unclear whether the protective effect of BHT can also be found in fully differentiated human neurons.

Ferroptosis has previously been implicated in the pathogenesis of AD (Wang et al. 2022; Yan and Zhang 2019; Ma et al. 2022) and our data suggest that ferroptotic cell death may also occur in the rat streptozotocin model of AD. Unfortunately, there is no specific histochemical method that selectively stains cells that undergo ferroptotic cell death, and thus, our conclusion is somewhat circumstantial. However, if one summarizes our findings the most probable explanation for our findings is that ferroptosis is a mechanical element in streptozotocin-induced neuronal dysfunction.

## Conclusion

A large number of preclinical studies suggested that anti-oxidative therapy might slow down the development of symptoms in human neurodegenerative diseases. BHT is a powerful antioxidant that has frequently been employed as additive in lubricants and cosmetics (Yehye et al. 2015). In this study, we tested the impact of oral BHT administration on the development of AD-related symptoms in an in vivo animal model of human AD. We found that oral BHT administration slowed down the development of AD related symptoms, reduced Aβ plaque formation in the CA1 region of the hippocampus and attenuated the expression of the App gene. Moreover, BHT reduced the degree of oixdative stress, limited neuronal iron overload and normalized the expression of ferroposis related genes. In the future, the effectiveness of BHT in other models of neurodegeneration should be tested.

## Data Availability

The original experimental raw data can be obtained upon request from PF and SA.

## Abbreviations

AD: Alzheimer’s disease
Aß: Amyloid beta
APP: Amyloid precursor protein
NFT: Neurofibrillary tangles
ROS: Reactive oxygen species
MDA: Malondialdehyde
BHT: Butylated hydroxytoluene
STZ: Streptozotocin
Y-maze: Y-shaped maze
GSH: Reduced glutathione
GSSG: Oxidized glutathione
GPX: Glutathione peroxidase
TBA: Thiobarbituric acid
PBS: Phosphate-buffered saline
H&E: Hematoxylin and eosin
DAB: Diaminobenzidine
PFA: Paraformaldehyde
NADPH: Reduced nicotinamide adenine dinucleotide phosphate
EDTA: Ethylenediaminetetraacetic acid
EGTA: Ethylene glycol-bis (β-aminoethyl ether)-N, N,N’,N’-tetraacetic acid
GR: Glutathione reductase
DTN: 5,5’-Dithiobis (2-nitrobenzoic acid)
HPLC: High-performance liquid chromatography
CA1: *Cornu ammonis* 1 (subfield of the hippocampus)
RNA: Ribonucleic acid
IL4: Interleukin-4
IL13: Interleukin-13
HDL: High-density lipoprotein
